# On my critical understandings about ‘modern epidemiology’

**DOI:** 10.1007/s10654-022-00944-8

**Published:** 2022-12-10

**Authors:** Olli S. Miettinen

**Affiliations:** (deceased) Faculty of Medicine, McGill University, Montreal, Canada

## Abstract

Editor’s Note: This Essay is the last paper of Olli Miettinen. I discussed it with him until about a week before his death on November 24, 2021. The article had gone through many iterations, as was the case with all his contributions to the European Journal of Epidemiology over the years. As Miettinen writes, it can be viewed as his academic mini-bio. Albert Hofman

The Editor-in-Chief of this journal, Albert Hofman, recently urged me, again, to write my autobiography, as a book. While reluctant to so write about myself, I am happy — keen, even — to write about matters academic on which I have something to seriously say to my readers. So, as a semblance of my academic mini-bio, I here sketch the evolution of such avant-garde understandings of mine about ‘modern epidemiology’ as I believe many readers of this journal should learn. Presentation of these in their evolutionary contexts facilitates the learning.

I urge students of epidemiology and my fellow scholars of the field to here read about these understandings as counselled by Francis Bacon; that is, “not to contradict and confute; nor to believe and take for granted; … ; but to weigh and consider” [1]. For I believe that much more common, thoughtful internalization of them would substantially advance teaching of research for the scientific knowledge-base of medicine, clinical medicine in particular — and could even advance the advent of the major reformation that is needed in medical education.

In lieu of an actual Abstract, I offer upfront a tidbit of the understandings I’ve reached. I say, *I’m not an epidemiologist, nor is or was any other purported ‘modern epidemiologist’.*

## My disappointments with education in ‘medicine’, and in ‘epidemiology & biostatistics’

I graduated with an *MD-equivalent degree* from the University of Helsinki in 1962. This provided for authorized practice of medicine, but *did not signify competence* in any of the disciplines to which medicine had splintered. That education covered an incoherent melange of subjects from some sciences and some disciplines of medicine. The Finnish term for this gemisch translated to ‘pharmascience’ — science! — in English. (It still does.)

No justification was given to that education as the *required propaedeutic* for education (and training) specific to whichever one of the disciplines that were viewed as medicine.

Among these were even some laboratory disciplines (pathology and radiology, i.a.) but not dentistry.

There was no express coverage of what I call the medical common — the concepts and principles that are relevant to all of the constituent disciplines of true medicine; that is, the *general theory of medicine*. We all should have been able, I thought, to study this as the truly-relevant — and vastly shorter — propaedeutic for studies in our chosen disciplines.

In the last semester of these studies we invited some non-academic doctors to teach us some real (rather than ‘academic’) medicine. I invited a dermatologist, Olavi Kilpiô. He began by saying, “I know you’ve had a semester of dermatology but don’t know dermatology. You’ve given me an hour-and-a-half; so I’ll teach it to you”. He began with the point that skin is either wet or dry, and either hairy or hairless. For each of the four types of skin he outlined the types of anomaly in it, and what to do in the face of each of these.

The *essential futility* of our studies for the MD-equivalent degree was my theme as an invited speaker (from abroad) in a reunion of my classmates. They privately expressed their agreement with this, but added that uttering it publicly could hamper their careers.

Thus disappointed, seriously, with my studies for that MD-equivalent degree, I commenced, in 1962, studies for a Master’s degree in *public health* at the School of Public Heath of the University of Minnesota — with *epidemiology* my ‘major’ and *biostatistics* the ‘minor’.

For these studies, different from those in the medical school, there actually was a *master textbook* [2]. It was a tome of near-1,600 pages. Nowhere in this humongous oeuvre was public health defined, nor could this term be found in its Index, even though it was in its title.

Of its nine sections, number eight was entitled Methodology; and there, as the first of its three chapters, was *epidemiology* addressed. It was defined as “that field of medical science [sic.!] which is concerned with the relationships of the various factors and conditions which determine the frequency and distribution of an infectious process, a disease, or a physiological state in a human community”. This definition (remarkably verbose and convoluted) of epidemiology (as a methodology-cum-science) left me mystified; for I felt it was disingenuous.

The ensuing chapter, entitled Statistical Reasoning, was said to be about “the nature of the reasoning process underlying the application of [the statistical method] in the study of man and his response to his total environment”. The word ‘*biostatistics’* was nowhere to be found in this omnibus textbook of public health, even though there was a Department for it at that SPH.

The remaining, third chapter under “methodology” covered “some of the knowledge of the health of the populations which has been gained through [the statistical reasoning addressed in the preceding chapter]” — as though this were a matter of methodology.

The *operative definition of epidemiology* in those MPH studies actually was one attributed to the School’s then Dean, Gaylord Anderson: “the science [sic.] of the occurrence of disease”[3]. But the mystery about its true essence persisted to me, as no note was taken of others’ views of it, even though they were being debated in the American Journal of Public Health.

That debate, sans resolution, led me to write my MPH thesis on its subject. Following some quotes from it I wrote that, “This confusion calls for sincere efforts to hasten the advent of the time when the essence and scope as well as the method of epidemiology are matters of knowledge rather than subjects for arbitrary personal opinion, debate, and joking”.

The statistical theory of research in that purported ‘occurology science’ was not meaningfully addressed in the MPH education on epidemiology & biostatistics. I therefore felt the need to supplement my MPH studies with those for a *Master’s degree in biostatistics.*

Those studies I began with the 1964 summer courses on it at the SPH of the UNC. Most notable in these was, to me, Colin White’s precept that, “One must be patient with mathematical statisticians; for they too are God’s children”. Returning, I promptly subscribed to a full-year course on ‘mathematical’ statistics. It was taught by Bernard Lindgren, with his “Statistical Theory” as the textbook. In no way did it try my patience. The rest of the ‘biostatistics’ education covered the statistical theory of subjects such as sample surveys (à la William Cochran), agricultural experimentation (à la Ronald Fisher), and bioassay (à la David Finney) — but not any statistical theory of epidemiological research!

I was, thus, deeply disappointed also with my studies of ‘epidemiology & biostatistics’.

## My discomforts with the fundamentals at the apex of epidemiologic academia

Then, after an interim year of studying multi-institutional trials on clinical interventions — under Christian Klimt at the University of Maryland — I took on appointment, in 1966, as Assistant Professor of Epidemiology and Biostatistics at Harvard School of Public Health. The invitation to this came from the then paragon of epidemiologic academics, *Brian MacMahon*.

From his textbook [4] I had learned his definition of epidemiology as “the study [sic.] of the distribution and determinants of disease prevalence [sic.] in man”. And I had also deciphered the meaning of this: ‘Study’ meant *science* again (rather than subject of learning, as in my ‘study’ of epidemiology). ‘Prevalence’ was a unique (and odd) construct related to *rates of occurrence.* ‘Disease’ meant any *illness* — defect and injury in addition to disease. ‘Distribution’ referred to the object of “descriptive epidemiology”, which “might be considered an extension of demography to health and disease”. ‘Determinants’ were characteristics of people, behavioural as well as constitutional and environmental, addressed in “analytic epidemiology”. And ‘man’ meant populations of people. In loco, I was pleased to learn that he had abandoned what he in that book presented as measures of the occurrence of illness.

The *operative concept* of epidemiology to MacMahon I had presumed to be the “analytic” version of it; and in loco I understood this to really be the case. By this he meant, as I had presumed, research on the (rates of) occurrence of illness *in relation to determinants* of this, causal relation in particular (and not any study of the determinants in themselves).

Central in the *“methods”* of “analytic epidemiology” was the duality constituted by “cohort” and “case history” studies. It had been adduced, I knew, by A. Bradford Hill together with Richard Doll, by their (rather capricious) decision to study the smoking etiology of lung cancer in a novel way, by means of follow-up of a cohort (of British doctors), classified at ‘baseline’ according to smoking habits. This new type of etiologic study they dubbed ‘prospective’, and the previously singular one ‘retrospective’. MacMahon used the term ‘case history’ in lieu of that ‘retrospective’, and he later adopted the more commonly-used ‘case- control’ term. Curiously, the word ‘etiology’ — for causal origin, etiogenesis, of illness — did not appear in that landmark book on etiologic research.

I initially retained, quietly, my prior reservations about both of these “methods”. But gradually I reached secure understanding of the *serious flaws* in each of them — together with understanding the *singular structure* of all well-construed studies for etiologic explanation of rates of morbidity. This understanding I here sketch in Sect. 6.

In the Department of Biostatistics of the HSPH, as in that of the SPH of the U of M, there was no express definition of that discipline; and it, too, had no program on the theory of statistical- type epidemiological research. This added to my disillusionment with ‘biostatistics’. It clearly wasn’t a logically-construed species of the genus statistics.

But I did complete the work for a PhD in this field from the U of M, in 1968. The thesis was on the theory of *matched non-experimental studies on causation*. Based on it, I published two articles in Biometrics. But: I later wrote a manuscript arguing that, actually, matching does not enhance the validity of those studies at all, nor does it ever optimize their efficiency; that *matching does not have any raison d’être* in them. That essay was rejected, unceremoniously, by the American Journal of Epidemiology; and I left it at that. Then, later yet, I gave a lecture on this tenet of mine as a guest of Noel Weiss at the University of Washington, to an audience of some 300. They wanted, and got, a full hour of discussion. And they evidently really learned the point of my epiphany — belated by my having taken the common teachings for granted.

In 1970, MacMahon assigned me to follow Jane Worcester as the teacher of *“the advanced course on epidemiology”.* That course had been, and remained, one of *epidemiology in the meaning of statistical-type epidemiological research.* By it being “advanced” I chose to mean that it was *introductory with advanced understanding of the fundamentals.*

There was much to advance in the theory of that type of research, from its fundamentals on up. The lecture notes I updated each year, as I went along. And even though they were intended for the students alone, they were being studied in a number of places. Most notable was their study in the Department of Biostatistics of the SPH of the UNC, where their study had the consequence that *epidemiology in this meaning of it was adopted as the focus of biostatistics!* David Kleinbaum in particular made a career of this *‘modern epidemiology’.*

But truth be told, *I remained uncomfortable about having become a party to this representation of epidemiological research as epidemiology* — as though epidemiology were a science, just as all of medicine purportedly was. But I remained reticent about this, too — lacking the courage of my contrarian-type views and convictions as a teacher even, just as before.

## The ‘consensus’ about the essence of ‘modern epidemiology’ as a science

The International Epidemiological Association in the first, 1983 version of its *dictionary of epidemiology* [5] repeated the (hermeneutically challenging) omnibus definition of epidemiology of MacMahon et alii [4], and not their much more focused, operative conception of it (above).

But added to this was “the application of this study to control of health problems “. In the sixth, 2014 edition [6], the corresponding ‘definition’ was essentially the same, but with considerable, curious elaboration of that common definition’s meaning.

In these ‘definitions’, there was an attempt to fuse two distinct entities, a (purported) science and the art it serves. But *logically, the ‘proximate genus’ of epidemiology is either science or art or something else* but not any union of these.

“*Teaching epidemiology”* has been the leitmotif of another series of high-profile books since 1992, most recently in its fourth edition [7]. Its Editors have worked on this with the IEA and the European Educational Programme in Epidemiology, as well as with the publisher (OUP).

This series of books on “ epidemiology”, like the textbooks of it in my school-of-public-health environments [3, 4], have been predicated on the idea that statistical-type research for (the knowledge-base of) community-level preventive medicine is epidemiology. And *journals of “epidemiology”, also*, have been ones of such research.

There thus has emerged — sans critical discussion — *apparent consensus* about the essence of “modern epidemiology” [8] as a science. For this, to-me-mysterious, aura of science about epidemiology — and about its mother, medicine — I’ve found what I believe to be its *extrinsic, furtive basis*. I came upon it serendipitously, once having relocated to the Faculty of Medicine of McGill University, in the mid-1980s.

## The extrinsic basis of the presentation of ‘modern epidemiology’ as a science

At McGill I soon realized that I had arrived where *William Osler* had grown ready to assume a status in medicine comparable only to those of Hippocrates and Galen. This status became established once he relocated to the then-nascent Johns Hopkins University. There, he begun with writing his epoch-making textbook of modern, *science-advanced medicine* [9]. And then, in 1893, he introduced a program of exceptionally *‘scientific’ education in medicine* there.

That education, together with the practice of medicine in the university’s hospitals, inspired Abraham Flexner — in his epochal report of 1910 [10] — to declare *modern medicine as a science*, “part and parcel of modern science”. He gave no compelling rationale for this. (He was neither a physician nor a scientist, but an ‘educator’.)

The American Medical Association, which had instigated the survey Flexner reported on, did not question that novel portrayal of medicine as a science. Instead, it made the Hopkins- type, science-heavy education the standard in the country at large. Medical faculties everywhere have followed suit. And they, as well as medical journals, have treated the nonexistence of tenable rationale for that Flexnerian claim as a non-issue, like a *taboo.*

The *unspoken truth* is that portrayal of modern medicine as a science boosted the social status of physicians and served as the basis for the profession’s authority and autonomy / sovereignty [11]. Medical academics, too, have been motivated to present medicine as a science, as though it obviously were [12]. And science-focussed ‘medical’ journals, too, have a vested interest in keeping the true basis of that false claim sub rosa.

Given that modern medicine at large is now so universally — though unjustifiably — portrayed as a science, the apparent consensus about modern *epidemiology* being a science can be seen to flow from this. A genuine consensus about that tenet would emerge from critical weighings and considerings of its rationale — from public discourse on it.

## Work on a pandemic illustrating the true, non-scientific essence of epidemiology

The Covid-19 pandemic is a source of cogent lessons on *the true, non-scientific essence of epidemiology*, and on its relation to science (in modern times).

*Lesson 1.* The students in introductory courses on epidemiology are, now, well able to recognize Covid-19 as an (important) object of *medical care* — by physicians — not only on the level of individuals but also *on the level of populations.* They appreciate that on the population level this illness has the (‘emergent’) properties of the frequencies — rates — of its occurrence, of *morbidity* from it. That duality in the clients of doctors the students are well-prepared to understand as the basis for the (profound) distinction between *clinical medicine* and *community medicine*. And community-medicine doctors involved in the Covid-19 pandemic’s control the students have witnessed being spoken of as *epidemiologists*.

So, the students are well-prepared to internalize the most fundamental lesson here, namely that *epidemiology is commonly medicine —* a doctor’s practice of this in terms of teaching the community’s population and authorities about control of the population’s level of morbidity from the disease (‘*doctor’* being Latin for ‘teacher’). And the students can readily be taught the linguistic point that, by extension of meaning, doctors’ practice of morbidity control is epidemiology even if the level of morbidity from the illness at issue is quite ordinary — endemic — rather than exceptionally high — epidemic.

*Lesson 2.* The students understand that competent teaching (‘doctoring’ in this sense) in the practice of community medicine — of epidemiology, that is — draws from preexisting general knowledge, from its implications in the context of the particulars of the situation at hand. They thus understand that (the practice of) epidemiology is fundamentally different from research for advancement of the general knowledge-base of it. So they understand that *epidemiological research is not epidemiology but science —* not medicine but medical science — for the advancement of the epidemiological arts of medicine. And conversely, they understand that *true epidemiology — community medicine — is not science*, however closely it may be science-based (scientific in this sense).

These understandings bring the students to conformity with the teachings of Aristotle. For to him, medicine was a *technê* or what we call art or craft — and, thus, very distinct from abstract knowledge, *epistêmê.* Theory of medicine was taught in his Lyceum, but the art of it was not (as it wasn’t really a gentleman’s concern). And correspondingly, research for community medicine is taught in today’s epidemiologic academia, but the art of it commonly is not (as it isn’t really a scholar’s concern). Just as that theory of medicine was not portrayed as medicine; *epidemiological research should not be portrayed as epidemiology.*

*Lesson 3.* The students have heard, and read, a great deal about *research* bearing on the pandemic’s control — about the laboratory-level research — bio-medical — for the development of candidate vaccines against the disease, and the human-level research — statistico-medical — for the requisite knowledge-base of their use.

The students would understand that familiarity with the research-and-development leading to the vaccines’ production and availability is not relevant for the epidemiologists to know about. And they would understand, also, that for competent teaching the policy-makers about the vaccines’ effects, relevant to know and understand only are scientific experts’ syntheses of the results of the relevant studies and their inferences from these.

## Notable falsities in the fundamentals of teaching epidemiological research

Apart from the overarching falsity that epidemiology is a science, notable others also commonly spoil teaching of statistical-type epidemiological research (etiologic).

Fundamental to this teaching naturally is *the concept of etiology.* Remarkably, this word was absent from the historic book by MacMahon et alii [4], as I already noted. The recent IEA ‘definition’ of the concept [6] opened with the claim that it “literally” is the science of causes — falsely implying that the ‘-logy’ postfix in a word always denotes science. (Tautology, e.g., is not a science; and “the science of causes” is nonexistent.) I defined etiology (as an aspect of illness) in Sect. 2 (as causal origin, etiogenesis).

In addition to the concept of etiology, *the essence of etiologic studies* should be correctly understood by teachers of “epidemiologic methods”. But it hasn’t been, either. In Teaching Epidemiology [7], in the chapter entitled “Teaching a first course in epidemiologic principles and methods”, said was that “a case-control study is a cohort study with an efficiency gain that comes from … ”. And the recent IEA dictionary [6] attached to its definitions of these two types of study the aside that a synonym for ‘case-control study’ is ‘case-referent study’. Both of these teachings were serious falsehoods.

In truth, the ‘case-control study’ is one in which a ‘case group’ of persons with the illness in question is compared with a ‘control group’ of persons without the illness, in terms of histories of some potentially-causal antecedent(s).

Before explaining how the IEA dictionary was amiss in its presentation of ‘*case-referent study’* as a synonym of ‘case-control study’, I note that Alfredo Morabia in his history of “epidemiologic methods” [13] said nothing about it — but, only upon having, for a start, replaced the ‘case-referent’ term by ‘case-control’ in his reference to the seminal article —. Miettinen 1976; a Citation Classic, no less — on this type of etiologic study. And he then had ignored all the subsequent literature in which this avant-garde concept had been addressed.

So I need to explain, as though de novo, that — in profound contrast to the case-control study — the *case-referent* or *case-base* study on the etiology of an illness I structured in reference to an express *study base* of case occurrence. The series of cases of the illness in question occurring in the study base is coupled with a sample of it; that is, the case series of person-moments is coupled with a base series of these (from the infinite number of these in the study base). This pair of series, suitably documented, is used to document the rate (incidence density) of the illness in the study base — which is *the study result’s referent.*

Specifically, documented is the way the rate in the study base depends on the then histories of interest — conditionally on the set of confounders — this in the framework of a logistic regression model for the case-base ratio in the two series. While the concept of study base (as the result’s referent) was the pivotal novelty in this, and also the leitmotif of my 1985 textbook on medical occurrence research [14], it too was absent from Morabia’s book [13].

For such a study, the *source base* (of population-time) is a segment of the source population’s course over time. The study base within this is the segment of it in which the constituent person-moments represent the domain of the study’s object (incl. one of the contrasted histories). In the initial stage of this study, the case series is identified, and the base series is selected, from the source base; and both of these series are then reduced to ones that actually represent the study base.The source population is either of the cohort type or dynamic; but regardless, the actual study population is dynamic.

*Cohort* (L. *cohors*, ‘enclosure’) I’ve defined as a population in which a person’s membership is based on experiencing the membership-clinching *event —* which means that the membership is never-ending and the population thus is closed for exit (even post mortem). The logical alternative of this I’ve understood to be defined by a *state*, for the duration of this — which leaves this type of population open for exit and makes it dynamic in the sense o turnover of membership. To MacMahon et alii [4] cohort was “an identified group” of persons. To this the recent IEA dictionary [6] added that it is “followed or traced over a period of time”. MacMahon et alii didn’t address dynamic population, and in that IEA dictionary it was ‘defined’ as “A population that gains and loses members”. Logically, actions on a population (its identification, etc.) have nothing to do with the nature of it (incl. whether it is a cohort or dynamic).

The time has come, I say emphatically, to start viewing the original, ‘case-control’ study, and the subsequently-introduced ‘cohort’ study, as just the initial, flawed attempts at formulating a study of the etiology of an illness. Neither one of these ‘methods’ appears in my 1985 book on the theory of medical occurrence research [14].

## Teaching epidemiological research to students and teachers of clinical research

While that recent Teaching Epidemiology book [7] was presented as a guide for “teachers in clinical medicine” (as well as in “epidemiology”, and in “public health” besides), actually at issue in it was, as I noted, teaching epidemiological *research* on the etiology of illness. Implicit in this is the view that study of the theory of this research is, as such, relevant preparation for clinical research as well. In this, the editors failed to see the bigger picture.

The editors seemingly didn’t appreciate that *etiologic* research for epidemiology is paradigmatic for research serving only a relatively small segment of clinical medicine. This segment is the pursuit of knowing about a particular antecedent in the etiology of a particular case of illness. Such knowing I call *etiognosis.* And, as I explained in the section above, those editors evidently didn’t understand the essential theory of this class of epidemiological studies.

— the nature of *the* etiologic study (i.e., the case-referent or case-base study).

A type of epidemiological study that would be much more important to teach to clinical researchers addresses the *prevalence* of an illness as a descriptive function (logistic) of its determinants. This would be paradigmatic for studies for the knowledge-base of *diagnoses* in clinical medicine *—* in the form of *diagnostic probability functions* for defined domains of case presentation [15]. While diagnostic research has been of central concern to ‘clinical epidemiologists’, their teaching of it has been seriously misguided [15].

Fundamental to this segment of the teaching is *the concept of prevalence.* MacMahon et alli [4] were, as I noted, quite confused about this. The recent IEA dictionary [6] gave a (verbose as usual and) confusing ‘definition’ of it. I say that it has to do with the frequency of a *state* of being, rather than an event, and is the proportion of a series (finite) or aggregate (infinite, innumerable) of person-moments such that the state in question is present.

Closely related to this is epidemiological study of the *incidence* of a near-term outcome, such as the proportion of live newborns ending up with ‘neonatal death’, or the proportion of vaccinated persons exhibiting an adverse reaction to it. Epidemiological study of such incidence as a descriptive-and-causal function — logistic — of its determinants should be understood to be, and should be taught, as paradigmatic for clinical studies serving *short-term prognoses —* for studying prognostic probability functions for these, with choice of intervention among the determinants of the probability of the outcome at issue.

But, along with diagnostic research, most important in the teaching that experts on the theory of statistical-type epidemiological research can now provide to students and teachers of clinical research has to do with studies for *longer-term prognoses —* intervention-prognostic experiments (‘clinical trials’) eminently among them. For, once they’ve come to grips with the essence of *the* etiologic study (Sect. 6), they’re uniquely prepared for understanding and teaching the study of probability functions for longer-term prognoses — in the framework of ‘Cox regression’ having been superseded by ‘Miettinen regression’ [15].

While the former focuses on the ‘risk sets’ — each of them involving the person-moment of the outcome event’s occurrence together with the others in the study cohort’s cross-section at that moment in cohort time (since the membership-clinching event) — the latter involves coupling the case series with a referent, or base, series of person-moments representative of the study base (the population-time of the study cohort’s follow-up for the event at issue). From these two series the study produces a logistic function for the quasi-rate (involving the number of base probes in lieu of study population-time); and from this it makes, very simply, the transition to the actual incidence-density function — which implies the corresponding cumulative-incidence function and, thus, the (empirical, survival-conditional) cumulative- probability function for the prognoses, specific to points of prognostic time together with the prognostic indicators and the compared interventions [15].

As is evident from this, masters of the theory of epidemiological research have centrally- important teaching to offer to students and teachers of clinical research. They should, I hold, view this as their *main mission* as teachers. These teachings should supplant those by ‘clinical epidemiologists’, who’ve been oblivious to the central mantra in rational thinking about clinical research for the knowledge-base of clinical medicine — the need to to address *gnostic probability functions* for suitably-defined domains of client presentation [15, 16].

## Onward and upward from these understandings about ‘modern epidemiology’

In Sect. 1 I argued that the existing type of interdisciplinary education in medicine needs to be replaced by education in the *general theory of medicine*. I recently sketched its contents, and submitted the sketch to BMC Medical Education. Its Editor-in-Chief, Kelda Manser-Smith, vividly illustrated what I explained in Sect. 4 — by summarily relegating it to hoped-for oblivion [17]. That general theory I would now follow with the *special theory* of general primary- care medicine (‘general practice’), if I had a suitable collaborator for this.

*Teachers of epidemiological research* I see to now to be able to learn the true fundamentals of etiologic research (Sect. 6), and then those of the four types of study for the requisite knowledge-base of clinical medicine (Sect. 7).

And I, now, leave the understandings I’ve here presented for each of my *readers to personally weigh and consider —* as to their bearing on his/her own future work, whatever be its relation to epidemiology and/or epidemiological research.
